# Supplementary material for: Hamstring muscle architecture assessed sonographically using wide field of view: A reliability study
Source: PLoS One. 2022 Nov 10;17(11):e0277400. doi: 10.1371/journal.pone.0277400 (PMC9648755; doi:10.1371/journal.pone.0277400)
Supplement: Attachment — Submitted filename: PLOSONE_comments_for_the_authors.docx [file pone.0277400.s001.docx]

**Comments for the author(s)**

Thank you for the opportunity to review the paper “*Hamstring muscle architecture assessed sonographically using wide field of view: a reliability study*”. The purpose of this study was to assess the reliability of static wide field of view ultrasound to quantify the muscle architecture of the hamstring muscles: Bicep Femoris long head, Bicep Femoris short head, Semitendinosus and Semimembranosus. The authors recruited 20 male amateur athletes and performed ultrasound measurements on two separate occasions. They concluded that static wide field of view is a reliable ultrasound technique to quantify the architectural characteristics of the hamstring muscles. Although the manuscript is very interesting and well written, some concerns about the sample size need to be point out and additional information are necessary.

**General comment**

- Why only 10 participants were recruited? Do the authors have any information to support that this sample size has sufficient statistical power for this reliability study? I believe this information is very relevant, and it is the main point to increase (or decrease) the support for the conclusions reported by the authors.

**Specific comments**

**Abstract**

Lines 51 and 52: I suggest being specific for the population investigated in the present study (male amateur athletes).

**Introduction**

- No comment.

**Materials and Methods**

- Line 141 – Why only 20 participants? Do the authors have any information to support that this sample size has sufficient statistical power for this reliability study?
- Line 141 – Why only men? Is there some rationale for not include women?

***Statistical Analysis***

- Line 212 – Is “3,1” a typo? Or it is supposed to be the reference number 31?

**Discussion**

- There is a mix of references styles over this section. For example, “(18, 19, 22, 33, 34)”, “(13) (17)”, “Chleboun et al. (2001)”, “Timmins and colleagues (2015)”. I suggest using only one reference style accordingly PlosONE authors guidelines.

**Conclusion**

- I suggest being specific for the population investigated in the present study (male amateur athletes).
